# The cultural construction of “executive function”

**DOI:** 10.1073/pnas.2407955122

**Published:** 2025-06-30

**Authors:** Ivan Kroupin, Helen Elizabeth Davis, Emily Burdett, Agustina Bani Cuata, Vahumburuka Hartley, Joseph Henrich

**Affiliations:** ^a^Department of Psychological and Behavioral Science, London School of Economics and Political Science, London WC2A 2AE, United Kingdom; ^b^Department of Human Evolutionary Biology, Harvard University, Cambridge, MA 02138; ^c^School of Human Evolution and Social Change, Arizona State University, Tempe, AZ 85281; ^d^Institute of Human Origins, Arizona State University, Tempe, AZ 85281; ^e^Department of Psychology, University of Nottingham, Nottingham NG7 2RD, United Kingdom; ^f^Tsimane Health and Life History Project, San Borja, Bolivia; ^g^One Pencil Project, Opuwo 21001, Namibia

**Keywords:** executive function, culture, cognitive development, schooling

## Abstract

In cognitive science, the term “executive function” (EF) refers to universal features of the mind. Yet, almost all results described as measuring EF may actually reflect culturally specific cognitive capacities. After all, typical EF measures require forms of decontextualized/arbitrary processing which decades of cross-cultural work indicate develop primarily in “schooled worlds”–industrialized societies with universal schooling. Here, we report comparisons of performance on typical EF tasks by children inside, and wholly outside schooled worlds. Namely, children ages 5 to 18 from a postindustrial context with universal schooling (UK) and their peers in a rural, nonindustrialized context with no exposure to schooling (Kunene region, Namibia/Angola), as well as two samples with intermediate exposure to schooled worlds. In line with extensive previous work on decontextualized/arbitrary processing across such groups, we find skills measured by typical EF tasks do not develop universally: Children from rural groups with limited or no formal schooling show profound, sometimes qualitative, differences in performance compared to their schooled peers and, especially, compared to a “typical” schooled-world sample. In sum, some form of latent cognitive control capacities are obviously crucial in *all* cultural contexts. However, typical EF tasks almost certainly reflect culturally specific forms of cognitive development. This suggests we must decide between using the term EF to describe 1) universal capacities *or* 2) the culturally specific skill set reflected in performance on typical tasks. Either option warrants revisiting how we understand what has been measured as EF to date, and what we wish to measure going forward.

## Significance

“Executive function” (EF) refers to a suite of cognitive control capacities, typically assumed to be universal. However, EF measures have not been developed and deployed universally. Rather, data on EF development come almost exclusively from “schooled worlds”–industrialized societies with universal schooling. We report comparisons of performance on typical EF tasks between children from schooled worlds and rural, nonschooled communities. Results show profound, sometimes qualitative, differences in performance, indicating typical EF tasks measure culturally specific skills, in addition to universal capacities. The term EF, then, can describe universal capacities *or* culturally specific performance on typical tasks—but not both. Either choice warrants revisiting how we interpret existing data from EF measures, and theories/measures of EF going forward.

Does “executive function” (EF) refer to universal features of the human mind? Or does it pick out something tied to a culturally specific set of experiences? Determining the extent to which each possibility holds true is essential for our understanding of a central construct in cognitive science. Defined as a family of cognitive mechanisms involved in the top–down control of behavior, EF has been, for decades, a major area of research on human cognition (1). EF tasks are also often used as controls or predictors in studies of other cognitive mechanisms (e.g., refs. 2 and^3^). These same tasks have been implemented in nationwide policies as normative assessments (4) and even argued to be a “culturally universal index of early development” (5).^*^

## Do We Have Evidence that EF, as Measured, Is Universal?

The widespread use and promotion of typical EF measures makes it all the more concerning that their presupposed universality has not actually been empirically confirmed [in line with the general Newtonian bias in cognitive science to assume universality despite insufficient evidence, 6). Specifically, EF research has, from the outset, discussed performance on typical EF measures as reflecting some universal cognitive control capacities, and described ontogenetic changes in performance on these tasks as evidence for typical patterns of human cognitive development (see ref. 7 for a historical review and, 8 for a dissenting opinion which highlights the role of domain-specific skills). At the same time, we simply do not have sufficient evidence to claim that whatever is measured by EF tasks is a universal cognitive feature. Indeed, as we review below, our understanding of EF is based almost entirely on data from standard tasks run in a specific set of cultural contexts, namely industrialized contexts permeated by the influence of formal schooling. In these contexts, children are raised from birth by adults whose parenting style has been shaped by formal schooling (9). This is followed by a dozen or more years of schooling during critical periods of cognitive development, and then life and work in environments where school qualifications and school-based skills are crucial for employment and daily function. We will refer to such industrialized, urban contexts permeated by formal schooling as “schooled worlds.”

Thus, if we set aside *definitions* of EF, the *data* to which the term refer do not discriminate between: 1) EF as a set of cognitive capacities universal across our species (such as the abilities to speak or walk) and 2) features of cognition particularly relevant to and/or powerfully shaped by the experience of schooling and/or life in schooled worlds (such as literacy or calculus).^†^ That said, worries about the cultural-specificity of what we discuss as EF may seem misplaced. For instance, it is demonstrably true that *some form* of top–down cognitive control exists across a wide range of species (10). Moreover, work on human EF appears to support universality in our species: Patterns of performance on standard EF tasks (which tend to be analyzed as a series of related factors, e.g., working memory, inhibition, and set shifting 11), have been replicated widely (e.g., refs. 1, 12, and 13). Furthermore, performance across tasks tends to correlate and is highly heritable (14). Obradovic et al. (5) provide a recent, explicit argument for the universality of EF (tests) on similar grounds.

## Evidence that EF Tasks May Reflect a Culturally Specific Skill Set.

A closer examination of the available data, however, complicates universalist claims based on data from schooled worlds. First, when we use the term EF we refer to a body of evidence that shows a “nearly slavish devotion to the use of EF tests as the sole or gold standard for its evaluation” (7). EF tests, in turn, are almost without exception structured around manipulating arbitrary, decontextualized information. To illustrate, consider two ubiquitous measures of EF development: 1) the *Dimensional Change Card Sort* (DCCS, 15) task, in which children must sort arbitrary stimuli first by one dimension (e.g. color) and then switch to sort the same stimuli by another dimension (e.g., shape), and 2) *backward span tasks* in which children are required to remember an arbitrary list of words and recite it back in reverse order. These are among a canonical list of developmental tasks compiled by Carlson (16), all of which involve arbitrary rules and/or decontextualized contents. The same is generally true of adult EF tasks (see e.g., ref. 17 for a list of adult tasks).

This property of EF tasks would not pose a problem for the assumption that performance on such measures reflects universal capacities *if* this type of decontextualized and arbitrary processing were equally required across all human populations — both in the contemporary world and throughout our evolutionary history. Yet, over half a century of cross-cultural research has emphatically demonstrated that this is not the case. Rather, performance on decontextualized, arbitrary tasks is demanded specifically and disproportionately by *those schooled worlds from which almost all EF samples are drawn* (see e.g., refs. 18–26). Gaskins and Alcala (27) provide a thorough discussion of these issues—focusing on the relationship between typical EF tasks and the patterns of task performance emerging in response to the demands of schooled worlds, in contrast to those of rural, nonschooled environments (see also ref. 28 for a more general review of cultural effects on EF task performance).

The uneven distribution of demands for decontextualized, arbitrary processing across populations highlights a terminological issue: As we review below, nonschooled groups tend not to give canonical responses on typical EF tasks. However, given the tight association in the literature between EF task performance and normative cognitive development (up to and including in national standards), describing such performance as “failures” makes it all too easy to slip into an inference that these groups lack some normative form of cognitive development. To avoid this presupposition, we will frame differences in performance in neutral terms, describing the patterns of behavior directly where this is feasible and referring to “canonical” (“success” as defined by the original task-setters) v. “noncanonical” responding otherwise.^‡^ We return to the question of what performance on these tasks may reflect in our discussion.

**Fig. 1.**
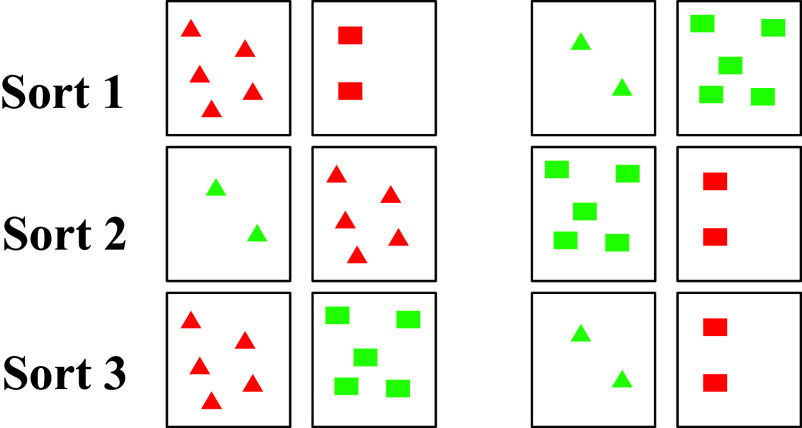
Card-sorting task, recreated from description by Gay and Cole (30). Participants were given four cards and asked them to sort along one dimension, then another then another.

Returning to the nature and scope of the effects of schooled worlds, consider an illustrative example from a card-sorting task: Participants are given a set of cards containing geometric shapes varying on three dimensions—shape, color, and number—and asked to sort them all according to one dimension (e.g., by color) and then re-sort according to another dimension (e.g., shape) and then according to the final dimension (e.g., number, Fig. 1). This task is nearly identical to the Flexible Item Sorting Task (29), a standard measure related to DCCS. Results with this task show that the EF capacities required for flexible sorting usually develop by age five in the schooled populations typically studied in cognitive science.

In contrast, working among unschooled Kpelle *adults*, Cole and Gay (30) report that while all their participants understood the task and successfully sorted the cards according to a first dimension (with individuals using different initial sorting dimensions), 35% did not make a second sort *despite unlimited time and explanations*, and 84% did not make a third sort. To drive home the point, consider that the Kpelle farmers in this study were typical, well-adjusted adults who were entirely functional in their own society. Their lives had simply offered *no need to develop* proficiencies involved in arbitrarily re-sorting cards.

## There Are Almost No Data on EF Task Performance Outside of Schooled Worlds.

Of course, we are not the first to identify a potential role for cultural context in the development of EF (13, for reviews see refs. 31 and 32) and a great deal of work has been done on the effects of formal schooling on decontextualized task performance more generally (see e.g., refs. 20, 21, and 26 for reviews). This wealth of evidence on other tasks emphasizes the paucity of data from outside of schooled groups on typical EF tasks: Even among cross-cultural studies of EF, research outside of typical, schooled worlds is extremely limited and data on the differences between schooled and nonschooled groups is essentially nonexistent. To illustrate, consider the most comprehensive list of work on EF tasks in which cultural effects of *any* kind were assessed, compiled by Schirmbeck et al. (32). The authors identified 26 studies which compared cultural samples on some measure of EFs, all of which were published between 2006 and 2018. Of these, 15 (58%) focused on comparisons between US or UK samples with samples from East Asia (China, Japan, Vietnam), conducted in schooled worlds, typically focusing on reasons for an observed advantage of East Asian children on EF tasks.

Of the remaining 11 studies, eight drew all of their samples from other schooled worlds (i.e., industrialized nations with universal schooling). Only three studies examined EF in contexts with limited formal schooling (33–35). Of these three, two collected samples from groups in which 75 to 90% of the population was literate (33, 34). The third collected samples from a periurban population with unspecified, but nonzero, levels of formal schooling (35). Thus, a review of the literature reveals fewer than 30 total studies on the effects of culture on EF. Of these, 23 (88%) sample participants from schooled worlds, all but one (97%) sample with a greater than three-quarters literacy rate and *all* of them sample populations with at least some schooling exposure.

Consistent with a role of schooled worlds on the development of EF as measured by standard tasks, the only study comparing a population with limited schooling to one with high-quality schooling showed qualitative differences in performance on typical EF tasks in the former groups. Legare et al. (35) report reliable development toward ceiling of switching between dimensions of matching when asked to do so on a variant of DCCS across the ages of 4 to 6 y, children with limited exposure to schooling showed *no increase* in the likelihood of switching across this age range, with children at all ages in this sample overwhelmingly not giving the canonical switching response. Pilot work from two other studies (neither of which made comparisons across groups) show similar patterns: A study by Obradovic et al. (5) using typical EF tasks, including DCCS, again found very low rates of canonical responses, as did Gaskins and Alcala (27) in a pilot sample of nine unschooled Yucatec Maya children.

In sum, the evidence to date is consistent with the possibility that EF, as we have been measuring it, is culturally specific and plausibly tied to life in schooled worlds. However, we have *no data* on *any* typical EF task performance in groups of children with no exposure to industrialized contexts or formal schooling whatsoever (i.e., in the state of humans for the overwhelming majority of our species’ history). This means that the data we refer to when speaking of “EF,” including when making normative claims about cognitive development, are ambiguous regarding the extent to which they are driven by universal or culturally specific factors.^§^

## Experimental Approach.

To the extent that EF tasks measure cognitive capacities that are universal across our species, performance on these tasks should be comparable across human groups. In contrast, if task performance is driven by exposure to specific cultural contexts, we should expect significantly, even qualitatively higher rates of canonical responding for children attending school and, in particular, those living in a schooled world.

With that in mind, we administered a battery of standard EF tasks to children from four populations, summarized in Table 1. We included a deliberately wide range of ages given previous results (such as those by Gay and Cole, 30) showing that canonical performance is not always observed even in adults from groups with limited or no schooling.

**Table 1.** Populations from which samples were drawn

Group No.	Location	Ethno-linguistic Groups	Schooled world	Schooling	N (age range, *mean age*)
1	UK	N/A	Yes	Yes, high-quality	76 (3–15, 7.7)
2	Bolivia	Tsimane	No	Yes, limited	73 (5–17, 10.6)
3	Kunene	Himba, Twa, Zemba	No	Yes, high-quality	70 (6–18, 11.7)
4	Kunene	Himba, Twa, Zemba	No	None	64 (5–17, 9.7)

The Kunene region lies on the Western side of the border between Namibia and Angola. Procedures for determining age in Bolivia and Kunene samples are detailed in the *SI Appendix*.

This set of populations allowed us to examine the performance of children with different degrees of exposure to schooled worlds. Specifically, we compare the UK group (1) to their peers outside of schooled worlds (groups 2 to 4). We also compare the schooled Kunene group (3)—who are exposed to a crucial element of schooled worlds, but not to the broader industrialized setting—to their unschooled peers (group 4) and Tsimane children (group 2) with limited-quality schooling. (By “limited-quality schooling” we mean attending school but typically lacking a common language with their teachers, see refs. 36 and 37, and *SI Appendix* for further details). The two groups from the Kunene region (3 and 4) on the border of Namibia and Angola were drawn from the same set of closely related tribal populations. These comparisons allow us to address the core question of this work, namely, do typical EF tasks plausibly tap features of cognition which develop in all human populations? Or does data from these standard cognitive measures reflect culturally specific experiences?

## Ethics and Participant Recruitment.

This work was given ethical approval by the Harvard University Committee on the Use of Human Subjects (IRB). Verbal consent was obtained from all participants. Participants in Bolivia, Namibia, and Angola were recruited during visits to villages and schools, after approval from local community leaders. Participants in these locations were given small gifts in compensation for their participation (e.g., a packet of sugar, Vaseline, cooking oil, pens, pencils; see also *SI Appendix* for further fieldsite details).

Participants in the United Kingdom were recruited via convenience sample during a free public engagement event, called Summer Scientist Week, hosted by the University of Nottingham. During the event, children (aged 4-to-12-y) are invited to campus for a 1-d visit to engage in different activities where they learn about psychology such as areas of the brain, perception, and emotion. The main part of the event is participating in different research studies. Participants did not receive compensation for this task, but their attendance of the Summer Scientist Week more generally involved various gifts, prizes, and games. (Note that while attendees of this program may differ from typical UK schoolchildren, they are likely to do so in the same way that all participants in typical EF studies do, since typical cognitive science samples are drawn precisely from families volunteering to visit university campuses).

## Methods

We employed five of the most common EF tasks used in the developmental literature drawn from Carlson’s (16) canonical list, as well as Verbal Fluency (VF), another common task. Here, we give brief descriptions of each task, with full procedural details available in our *SI Appendix*. DCCS and *backwards span* we have already described above. *Forward span* is identical to backwards span but children repeat the list of words they hear in the same order that they hear it. *Luria’s hand game* involves the experimenter making a fist or an open palm gesture and the child repeating their gesture for 15 trials (training phase), after which the child is told they must make the opposite gesture to that of the experimenter (15 trials, test phase). VF requires participants to name as many items from a particular category (e.g., animals) as possible in a set duration (we used a duration of 2 min, some studies use one). Note that this kind of reasoning is likely not what many populations would consider to be VF. As such we will refer to it simply as the “VF task.”

### Ecological Validity and Task Demands.

We anticipate a number of important objections to our experimental approach. First, tasks developed in schooled worlds are unlikely to accurately reflect the capacities of other populations (e.g., refs. 38–40). Here, however, we are not interested in developing a calibrated measure of children’s competencies across our samples. In the absence of compelling evidence otherwise, it seems reasonable to assume that any group of children, including those in our samples, are *competent in their own environments*, presumably showing some normal distribution of relevant capacities and skills (see e.g., ref. 35). The present study concerns the nature of what we cognitive scientists have been measuring as EF—not the capabilities of children within their own cultural environments. As such, we are interested precisely in response variation across our four populations on measures which are key to our current understanding of EF, as opposed to selecting and modifying EF measures to support better performance in groups with limited or no schooling (as in, e.g., refs. 5, 22, and 23).

To ensure that participants’ performance did not reflect a failure to understand task instructions, or other irrelevant task demands (including motivation to participate at all, an issue highlighted by refs. 27 and 28), we deployed a variety of methodological checks to verify understanding in the case of every task and exclude participants who fail these checks. In some cases, this involves passing versions of the test task, versions which are identical in content and differ only in a crucial manipulation (i.e., preswitch or imitation trials in DCCS and Luria’s hand game). For other tasks (Forward Span, Backward Span, VF) these checks involve producing the canonical responses (i.e., recalling at least some words correctly in the span tasks, producing at least some items from the right category in VF).

Participants overwhelmingly passed comprehension checks in all tasks except for Backwards span (which we discuss separately, below): Pass rate was 90% or more in all groups for DCCS, 97% or more in all groups for VF and 100% for all groups for forward span and Luria’s game (see *SI Appendix* for details). The following results, then, should accurately reflect performance on precisely the kind of typical EF tasks which “the vast majority of studies published on the topic of EF have used… to determine whether certain disorders impaired EF or how EF developed in normal samples” (7). That is, we see no reason to believe differences in performance are due to children essentially misunderstanding the nature of the tasks (we also return to this issue in the discussion).

In all samples, children were tested by themselves, one at a time, in a separate space (e.g., empty classroom, large tent). In all cases, the experimenter took a few minutes to greet the child and explain that they would be playing a few games together. In no case were experimenters familiar to participants ahead of time. In these and other respects, then, testing closely resembled the typical deployment of EF tasks as they have been used in the literature—in line with our general strategy of studying what these tests have been measuring as conventionally deployed. Further details of the populations and our methodological approach are available in the *SI Appendix*.

## Results

Our results were consistent with EF tasks measuring culturally specific cognitive skills, not universal features of cognition as typically presupposed. Here, we report regressions, controlling for age and sex, comparing the four populations in our study on all tasks except for backwards span, which is discussed separately below. Tables 2 and 3 present the two key comparisons of interest here, i.e. between groups in and out of schooled worlds (Table 2, with group 1–the UK children–as a WEIRD reference category) and rural groups with and without high-quality schooling, i.e., a comparison between schooled and unschooled Kunene participants (Table 3, with group 3–the schooled Kunene sample—as a reference category). All other comparisons are reported in *SI Appendix*, which also links to our data and where we also display the data as scatterplots.

**Table 2.** Regressions, controlling for age and sex, comparing participants from a schooled world (UK, group 1) to their schooled Kunene (group 3), Bolivian (group 2), and unschooled Kunene (group 4) peers across four typical EF tasks

	Dependent variable			
	DCCS	Luria’s test	VF	Forward span
	*Logistic*	*OLS*	*OLS*	*OLS*
	(1)	(2)	(3)	(4)
Age	0.342^***^	0.335^***^	1.108^***^	0.081^***^
	(0.071)	(0.071)	(0.102)	(0.015)
Sex	−0.401	0.055	0.535	0.083
	(0.356)	(0.422)	(0.603)	(0.087)
Schooled Kunene	−2.071^**^	−1.606^*^	−13.130^***^	−1.238^***^
	(0.769)	(0.648)	(0.923)	(0.134)
Bolivia	−3.760^***^	−1.814^**^	−15.046^***^	−0.906^***^
	(0.693)	(0.609)	(0.875)	(0.126)
Unschooled Kunene	−4.118^***^	−3.074^***^	−14.848^***^	−1.376^***^
	(0.695)	(0.611)	(0.877)	(0.126)
Constant	1.006	9.366^***^	13.816^***^	3.483^***^
	(0.784)	(0.728)	(1.042)	(0.151)
Observations	267	283	279	283
Log Likelihood	−101.470	−754.973	−841.662	−308.968
Akaike Inf. Crit.	214.941	1,521.945	1,695.324	629.936

The type of regression is indicated in italics under the task name (logistic or ordinary least squares, OLS). *P < 0.05; **P < 0.01; ***P < 0.001.

**Table 3.** Regressions from the same model as Table 2 but with the schooled Kunene group (3) as reference category

	Dependent variable			
	DCCS	Luria’s test	VF	Forward span
	*Logistic*	*OLS*	*OLS*	*OLS*
	(1)	(2)	(3)	(4)
Age	0.342^***^	0.335^***^	1.108^***^	0.081^***^
	(0.071)	(0.071)	(0.102)	(0.015)
Sex	−0.401	0.055	0.535	0.083
	(0.356)	(0.422)	(0.603)	(0.087)
Unschooled Kunene	2.071^**^	1.606^*^	13.130^***^	1.238^***^
	(0.769)	(0.648)	(0.923)	(0.134)
UK	−1.689^**^	−0.209	−1.916^*^	0.332^**^
	(0.530)	(0.597)	(0.850)	(0.123)
Bolivia	−2.047^***^	−1.468^*^	−1.717	−0.138
	(0.534)	(0.629)	(0.896)	(0.130)
Constant	−1.065	7.760^***^	0.686	2.245^***^
	(0.806)	(0.953)	(1.368)	(0.197)
Observations	267	283	279	283
Log Likelihood	−101.470	−754.973	−841.662	−308.968
Akaike Inf. Crit.	214.941	1,521.945	1,695.324	629.936

The type of regression is indicated in italics under the task name (logistic or ordinary least squares, OLS). *P < 0.05; **P < 0.01; ***P < 0.001.

Across all four measures of EF, holding age and sex constant, children living in a schooled world (UK, group 1) were significantly more likely to produce canonical responses (e.g., switch in DCCS, recall more words in span tasks) than their peers from all groups on all tasks (Table 2, *Backwards span*, below). In other words, controlling for different distributions of age and sex across groups, our results show that participants from a schooled-world sample—i.e., the kind of participants which make up almost all data in EF research—show major differences from populations underrepresented in the literature. Furthermore, Table 3 shows that the schooled Kunene group (3) gave more canonical responses than their nonschooled peers (group 4) on all tasks, showing negative coefficients across the board, though the coefficient on VF and Forward span are poorly estimated (Table 3, *Backwards span*, below). Perhaps more strikingly, performance on some tasks showed qualitative differences between groups. We review results for each task in detail next.

### DCCS.

**Fig. 2.**
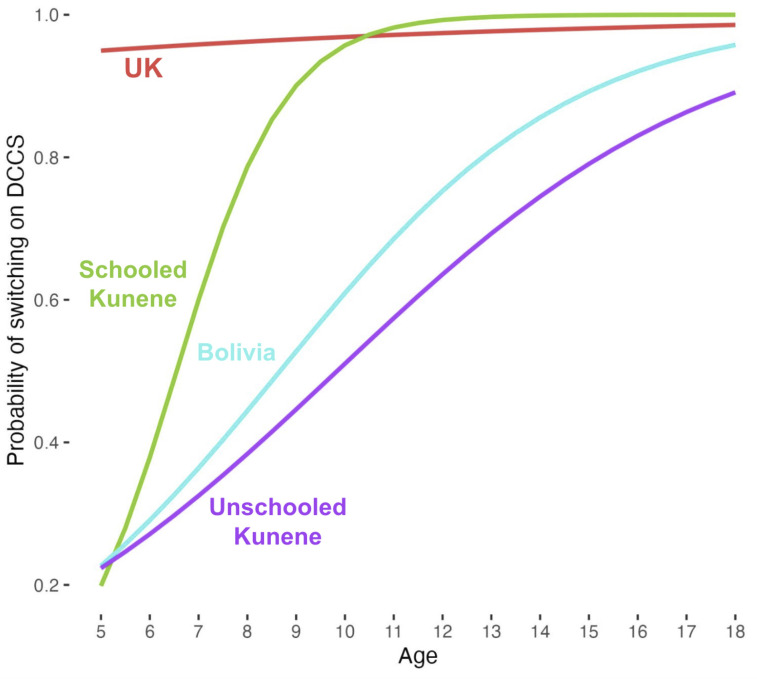
Logistic curves for probability of successfully switching on DCCS.

DCCS and its variations (12) are among the most commonly used developmental EF tests and have been found to show a step-change pattern across development, with almost all 3-y-olds perseverating (continuing to sort by the original dimension after being asked to switch) and almost all five-year-olds switching (15). Our results from the UK group replicate these findings, with almost no children above the age of five perseverating. The schooled Kunene sample was dramatically closer to the performance in the UK sample compared to either the Bolivian and unschooled Kunene groups: Converting logistic regression coefficients in Table 2 to odds ratios for interpretability, the schooled Kunene group was 7.9 times less likely to switch in DCCS than the UK group, the equivalent ratios for the Bolivian and unschooled Kunene groups were 42.9 and 61.4 times less likely, respectively. Fig. 2 displays logistic curves for DCCS performance in all four groups.^¶^

Another way of highlighting the difference in performance by children with and without high-quality formal schooling is highlighted if we consider the proportion of children perseverating *after the age of 10* (i.e., *double* the typical age of switching in samples from schooled worlds): No children in the UK sample perseverated after age 10, and only one child (out of 41, 2%) in the schooled Kunene sample did so. In contrast, 19% of Bolivian (6 out of 31) and 35% of unschooled Kunene children (7 out of 20) over age 10 perseverated on DCCS.

### Luria’s Game.

Clear population-level differences emerged in Luria’s game: The UK (group 1) children were much more likely to counterimitate the experimenter in the test phase than all of their peers (Table 2). The two groups with schooling exposure outside of a schooled world (2 and 3) did not differ in their performance on the task, while the unschooled Kunene group were less likely to counterimitate when instructed to do so compared to all other groups, despite comprehending the task (i.e., 100% understood instructions; see also *SI Appendix*).

### VF Task.

In the VF task, children in the United Kingdom named far more members of categories (e.g., animals) than their peers in other groups (Table 2), with the effect of living in a schooled world dwarfing any possible effects of schooling alone (e.g., the coefficient between UK and schooled Kunene is at least seven times larger than any coefficient between schooled Kunene and the other two groups, Table 3).

### Forward Span.

**Fig. 3.**
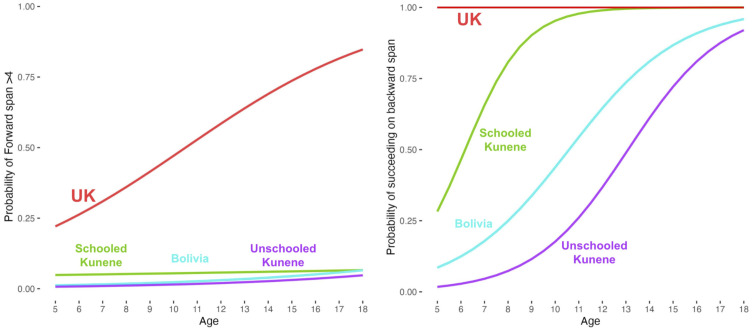
Logistic curves for probability of recalling more than four items on forward span (*Left*) and of succeeding at all on backwards span (*Right*).

In the Forward span task, the schooled samples recalled more words than their peers from outside schooled worlds (Table 2). The more striking pattern of data emerged from the latter three groups (2–4) when we consider the proportion of children able to recall more than four words back from a random list of common nouns: Out of a combined total of 207 children from groups 2 to 4 who understood the task instructions, *only seven* (3%) recalled more than four items (consistent with a human working memory limit of four items, when controlling for long-term memory and rehearsal, (41)). In contrast, 27 out of 76 children in the United Kingdom (35%) recalled more than four items—a discrepancy further highlighted by the fact that the UK sample had the lowest average age (Table 1). Fig. 3, *Left* shows logistic curves for participants performing below/above four items on forward span.

### Backward Span.

In all of the tests discussed so far, we report data only from participants who pass our comprehension checks (e.g., listing words from the requested category in the VF task, matching by the requested dimension in DCCS preswitch trials). This approach, however, proved inappropriate for backwards span due to the rate at which children without exposure to formal schooling did not reproduce any words from a list in reverse order when asked: While 100% and 91% of UK (group 1) and schooled Kunene (group 3) participants, respectively, recalled at least two words in a list in reverse order when asked to do so, 50% of Bolivian participants and 22% of unschooled Kunene participants did not. This was despite up to four repetitions of the instructions, with examples, for every participant. Fig. 3, *Right* displays the logistic curves for participants recalling even two items in reverse order.

A binomial logistic regression confirms that both age and location were significant predictors (χ^2^(1) = 50.267, χ^2^(3) = 147.567, respectively, *P* < 0.0001 in both cases) and Tukey post hoc tests revealed that schooled Kunene participants were more likely to be able to recite words in a reverse order than Bolivian and unschooled Kunene participants (*P* < 0.0001 in both cases).

## Discussion

Our results indicate that what psychologists have historically measured as EF in fact conflates latent, universal cognitive control capacities with culturally specific patterns of responding. While the causal role of schooling and schooled worlds on the development of these abilities remains to be confirmed, the data are consistent with this possibility: Children from the United Kingdom (an industrialized society with universal schooling) gave much more canonical responses than the groups outside of schooled worlds, and the schooled Kunene sample (partly exposed to a schooled world through high-quality formal schooling) often gave more canonical responses than their rural peers in Bolivia and Kunene with little or no exposure to schooling, respectively. We return to the question of what exactly these differences may reveal below. First, however, we briefly review the results for each task before addressing some alternative explanations and sketching further directions.

### DCCS.

Despite being a flagship measure of developmental EF argued to reflect prefrontal cortex development (42) and used to inform national policy on early cognitive development (4), the developmental pattern in DCCS of switching between dimensions of sorting by 5 y old emerges *only* in a sample drawn from a schooled world. The specificity of the arbitrary rule-switching performance to formally schooled contexts is evident through the dramatic difference between schooled and unschooled Kunene groups (Fig. 2 and Table 3). The alternative to such a cultural interpretation is to take work on DCCS at face value and assume that 35% of unschooled Kunene children *10 and older* (who did not pass DCCS) have not achieved the same prefrontal cortex development to Western *5 y olds* (who do in almost all cases). This is unlikely.

### Luria’s Game.

Despite the apparent simplicity of Luria’s game, with its back-and-forth repetition, living in a schooled world and attending formal schooling makes giving canonically correct responses more likely—again potentially due to the arbitrariness of the rules and switches. Notably, this was the only test on which the schooled Kunene group (3) did not reliably differ from our Bolivian sample (4), who were ostensibly also in school but received limited instruction due to pervasive language barriers. We cautiously conjecture that the habit of Bolivian teachers leading students in repeating back patterns banged out on desks may have contributed to the latter group’s performance in engaging with hand gestures by the experimenters.

### VF Task.

Children living in a schooled world (UK) produced many more category members than other groups in the VF task. However, there is a clear alternative hypothesis to this being a difference in EF skills: While we chose domains (e.g., foods, animals) for which for all of our samples would contain a large number of known words, it is possible, that children develop greater vocabularies for certain subjects in the process of formal schooling. This increased vocabulary may, in turn, account for the advantage in canonically correct responses of schooled over unschooled Kunene participants, and of UK participants over all other groups. Thus, while future work may do well to consider a vocabulary-controlled version of VF across a diverse set of populations, VF *as it has always been conducted* is manifestly not a measure of a universal ability.

### Forward Span.

In the Forward span task, children living in schooled worlds recall many more words than those from outside of schooled worlds. This pattern converges with neuroscientific evidence that the upper limit of human working memory, without meta-cognitive strategies such as rehearsal, is four (see ref. 36, *The Magical Number 4 in Short-Term Memory*). In conjunction, these results suggest (though do not definitely show) that the meta-cognitive strategies required to improve beyond four items in an arbitrary recall task *may not develop at all* without very specific cultural experiences, such as those provided by schooled worlds. In either case, typical forward span tasks quite clearly tap abilities which are significantly shaped by differences in cultural experience.

### Backwards Span.

In the Backwards span task, a large majority of participants without access to high-quality schooling did not repeat back words in a reverse order despite multiple examples and instructions and uniform success in recalling at least some words during the forward span task. Notably, the patterns of development across the four groups strongly resemble ones observed in DCCS (Figs. 2 and 3, *Right*, respectively). In both cases, performance evident by age 5 in schooled-world samples emerges with some years delay outside of schooled worlds if formal schooling is present, and slowly if at all when formal schooling is absent.

In sum, given that the data EF refers to come overwhelmingly from standard tests, the evidence we have presented here rules decisively against the term picking out a universal cognitive skill set. Rather, the present results indicate the developmental curves studied as EF in fact capture something culturally specific, potentially related to formal schooling and schooled worlds in particular.

#### Task performance vs. culturally specific skill.

If developmental trajectories on typical EF tasks do not reflect universal patterns of cognitive development, do they reflect *anything* generalizable and interesting? It may be possible (as suggested by some cross-cultural researchers, including a reviewer on this paper) that performance on typical cognitive tests may not reveal *anything* about enduring cognitive features or skills, rather reflecting *only* familiarity with certain testing contexts. One potential line of evidence for this position is that, in certain other decontextualized tasks, minimal amounts of pretraining or reframing of the task allow for much greater success in nonschooled participants. For instance, solving a syllogism problem can be overcome by something as simple as framing it in terms of an imaginary world—bracketing the participants real-world intuitions (e.g., ref. 43).

We believe that such a deflationary account is both empirically and theoretically unsupported. First, because at least some of the tasks used in this study (e.g., forward span) were clearly comprehended by participants, yet showed major differences in performance nevertheless. Second, because our work adds to decades of research which shows that performance on decontextualized, arbitrary tasks involves some specific set of prior expectations or skills which emerge differentially across populations—especially across those with and without exposure to formal schooling. In fact, given the well-documented and highly systematic differences between schooling and traditional learning contexts (e.g., refs. 18, 44, and 45) it would be surprising if there were no systematic and important changes to skills and dispositions between schooled and unschooled populations. Such changes have been shown to span the range from differences easily alterable by training (as in syllogism studies) to the qualitatively distinct (as in the case of backward span performance). We suspect that there are stable and important adaptations at all levels, and that even easily alterable dispositions play some role in facilitating performance in the individual’s cultural context (see ref. 6 for discussion). Note that “skill” here does *not* imply a normatively important form of cognitive development. Both juggling on a unicycle and emotionally manipulating your relatives are skills, but not ones that are universally useful or laudable. Thus, while it seems clear to us that population differences shown here are differences in some forms of disposition or skill, the nature of these dispositions and skills, and their utility in various cultural contexts, remains an open question. The latter especially so because we know of *no* studies relating EF task performance to life outcomes outside of schooled worlds. Further empirical and theoretical developments detailing the nature of these multilevel changes to disposition and skill, then, remain a key direction of future work. Whatever the results of such future investigations may be, however, they do not alter the fact that EF as it has been measured to date partially reflects a set of culturally specific dispositions and skills and not only universal features of the mind. We return to the question of how this affects our use of the term EF below.

### Alternative Explanations: Harsh Environments and Genetic Differences.

Before we draw our final conclusions, it is important to address two alternative explanations for the pattern of data reported here. First, it is clearly the case that forms of environmental harshness such as poor nutrition, unmanageable stress, or trauma can result in major cognitive delays (e.g., refs. 46 and 47). Second, group differences could be genetic in origin. After all, performance on EF tasks is clearly heritable, at least in some Western populations (14).

First, whether these factors may contribute to our findings is an important avenue for future work. We do not, however, see them as undermining the conclusion that EF tasks measure, in part, culturally specific skill sets. That is, it is logically possible for EF tasks to be measures of universal capacities and for the pattern of data above to reflect impairment in universally developing prefrontal functions in children outside of schooled worlds. If this is true, however, results from DCCS would indicate that 35% of unschooled Kunene children have more limited prefrontal cortex development at age 10 than their schooled UK peers at age 5. The implied, profound cognitive delay is incompatible with uncontroversial observations: Ten-year-old children in rural Kunene are routinely and effectively involved in many tasks which are incomparably more challenging than those faced by schooled-world five-year-olds, e.g., care for livestock, siblings, collection of food, firewood, and other key functions of their society. The same interpretative issue holds still more strongly in the case of qualitative differences, such as those found in the Backwards-span task. In these cases, the relevant cultural input seems to be wholly absent without exposure to schooling or schooled worlds.

Moreover, even if genetics or adverse environments account for part of the observed patterns, these impacts are orthogonal to the question of the extent to which a given skill is culturally specific. For instance, increasing starvation or the progression of Huntington’s disease will, eventually, prevent a cellist from playing. This, however, has no bearing on the fact that performing Bach concertos is a culturally constructed skill. Again, we are not suggesting that environments or genetics are irrelevant to life outcomes. Our point here is that the effects of genetics or environmental harshness cannot be so large as to support an alternative to EF tasks measuring culturally constructed skills. In any case, future work will do well to explore the interrelationship between such factors, effects of different cultural environments and the development of EF.

### Adapting Measures and Reexamining Constructs.

This work comes amid a surge of efforts toward developing EF measures which are sensitive to local contexts, including in nonindustrialized, nonschooled societies (e.g., refs. 5, 27, and 28). We see the present study as a complement to these efforts. After all, we will not be able to create better measures of EF without the kind of detailed evaluation of cultural differences provided in these studies (see e.g., refs. 22, 27, 48, and 49 for key examples of adjusting experiments to local cultural context). At the same time, the present results indicate that we must, in parallel, reevaluate what we are trying to measure in the first place.

To illustrate, consider the reverse case: A Himba psychologist may see cattle-herding as comprising a critical set of cognitive skills. These include, at a minimum, long-term attention simultaneously tracking the herd itself, its vulnerable or misbehaving members, environmental threats, position in the landscape, and so on. Pursuing cross-cultural fieldwork, she finds, to her surprise, that children in Boston are completely inept when given a small herd and asked to guide it across a day’s pasture. In order to construct a culturally appropriate task, she develops a herding video game, which the Boston children perform much better on. This adaptation may certainly give a better representation of Boston children’s skills of day-long diffuse attention by removing culturally unfamiliar elements (e.g., live goats). However, the fact remains that the task measures a skill set which is still culturally specific—and likely of limited use in a Boston life. After all, Boston children likely switch between discrete activities (playtime, nap time, preschool, being driven between these, etc.) every couple hours, if not more often, and have few if any responsibilities comparable to keeping a few dozen goats alive.

By no means are we suggesting that other authors have made this kind of crude mistake: Work on designing culturally sensitive measures of EF is typically far more subtle, addressing the issue of culturally relevant skills head on (see ref. 27 in particular). Here, we simply wish to emphasize that the adaptation of tasks must be paired with a reexamination of the constructs which the unadapted tasks were measuring in the first place. After all, our data indicate that performance on typical EF tasks depends on skills which, like the skills involved in herding, are not (equally) relevant in all cultural contexts.

### Summary and Future Directions.

Our data show that what psychologists have measured as EF is likely to include a culturally specific skill set—one whose utility outside of testing situations and/or formal schooling remains an open question. In particular, these results are consistent with, but certainly do not prove, experience with formal schooling and/or schooled worlds being particularly crucial to the development of what is measured by standard EF tests. Regardless, we see it difficult to avoid the conclusion that the relationship between diverse cultural environments and EF as we currently measure it warrants a great deal more investigation.

In previous work, we have referred to the joint-like interrelation between cognitive capacities and specific cultural environments as *articulation* (6). Our point here is that understanding cognitive capacities requires research not only on cognitive performance, but also on the environments with which cognition of different groups is articulated (e.g., schooled worlds, rural, nonschooled communities). In doing so, we build on a rich empirical and theoretical tradition focusing on the interrelation of culture and cognitive development which culturally located work on EF will do well to integrate (see e.g., refs. 39, 44, 45, and 50–54), for frameworks and reviews as well as 27 for an important effort on EF specifically).

Moreover, from the perspective of EF as an *articulation* between cognition and environment, the field plainly suffers from an absence of data on the environment side of the joint. After all, EF research rarely appeals to data concerning the nature of tasks which children face day-to-day, and how these vary across cultural context (though see ref. 27). The need for more relevant environmental data also aligns with critiques of EF from researchers working *within* schooled worlds, notably Doebel’s (8) recent call to reconceptualize EF in terms of context-specific, goal-oriented cognitive skills.

At the very least, we can no longer presuppose that EF refers *only* to a universal skill set. Rather, we appear to be faced with two alternatives: 1) We can continue using EF to describe performance on EF tasks (i.e., the vast majority of EF data to date). In this case, we either need to acknowledge that this term refer partly to culturally specific skill sets, or prove, contrary to what is suggested by the current results, that performance on typical EF tasks *does reflect* only universal features of cognition. 2) We can coin some new term for those cognitive-regulation skills tapped by EF tasks which are culturally specific, and continue to use EF to refer to some underlying, universal mechanisms (e.g., those potentially shared with other species) with the latter being required, but not necessarily sufficient, for typical EF task performance. Whatever path we choose, EF will need to be redefined relative to existing data from standard tests, and the broader constellation of terms relating to self-regulation and top–down cognitive control (see ref. 55 for a discussion of these interrelated constructs). Similarly, we see developing better accounts of the nature of culturally specific dispositions and skills related to EF task performance, as well as universal cognitive regulation capacities, as a key future direction of research.

Ultimately, there can be no doubt that healthy cognitive development, including cognitive control and self-regulation skills, are critical to life outcomes in *any* environment. Likewise, *if* our aim is to promote the kind of development specifically demanded by schooled worlds, typical measures of EF may well serve as important predictors of developing skills valuable within these environments (though see refs. 7 and 56 on poor correlations between EF tasks and real-world outcomes). However, schooled worlds represent only a fraction of human environments, both today and throughout our species’ history. With this in mind, it behooves us to develop and test theories which explicitly relate the skills demanded by EF tasks to empirical data on the demands and affordances of a broad range of human environments. The stakes are high, as cognitive science strives to define how we understand human cognition and, increasingly, informs the normative goals of national and global policy on education and child development.

## Supplementary Material

Appendix 01 (PDF)

## Data Availability

Anonymized .csv with full results data have been deposited in OSF (https://osf.io/8uz9s/) (57).

## Footnotes

^*^Notice that while EF belongs to a broader family of constructs related to cognitive control and self-regulation (55), we are concerned here specifically with the subset of this work which refers to EF.

^†^It has also been argued that EF*as a universal capacity* may lack coherence and ecological validity, with many measures correlating poorly with observational data (see e.g., refs. 7, 55, and 56). This critique is orthogonal to the problem we raise here, though it similarly motivates a reconsideration of EF as a term.

^‡^We thank an anonymous reviewer for highlighting the importance of explicitly clarifying these issues.

^§^In fact, work using non-standard measures of cognitive flexibility adapted to make decision-making more contextual and less arbitrary shows that predominantly unschooled adults actually *outperform* their Western, schooled peers (58). Such data, and the absence of such tasks from the body of evidence used to support EF is still another reason to explicitly test the presupposed universality of this construct.

^¶^Sampling across multiple locations in remote locations across a wide range of ages limits our power to explore age-by-location interactions in these regressions. Adding these interactions reveals the expected patterns, but the SE expand, in many cases making the coefficient estimates imprecise. Consequently we emphasize qualitative differences (e.g., teenagers not switching in DCCS, overwhelming lack of backwards recall of word lists) as evidence that EF as measured by typical tasks involves culturally-specific skills.
