# Supplementary material for: The cultural construction of “executive function”
Source: Proc Natl Acad Sci U S A. 2025 Jun 30;122(27):e2407955122. doi: 10.1073/pnas.2407955122 (PMC12260431; doi:10.1073/pnas.2407955122)
Supplement: Supplementary file 1 — Appendix 01 (PDF) [file pnas.2407955122.sapp.pdf]

Supporting Information for

The cultural construction of "executive function"

Ivan Kroupin[1,2,*], Helen Davis[2,3,4], Emily Burdett[5], Agustina Bani Cuata[6], Vahumburuka Hartley[7] and Joseph Henrich[2]

1-Department of Psychological and Behavioral Science, London School of Economics and Political Science; 2-Department of Human Evolutionary Biology, Harvard University; 3-School of Human Evolution and Social Change, Arizona State University; 4-Institute of Human Origins, Arizona State University; 5-Department of Psychology, Nottingham University; 6-Tsimane Health and Life History Project; 7-One Pencil Project

Corresponding author: Ivan Kroupin

Email: ivan@kroupin.com

This PDF file includes:

Supporting text
Tables S1 to S9

Other supporting materials for this manuscript include the following:

Dataset S1 - Available at https://osf.io/8uz9s/

R code S1 - Available at https://osf.io/8uz9s/

Supporting Information Text

Here we present details of methods and additional regression tables

Additional information on methods and subjects

General procedure

The EF battery was run in a fixed order across all children and all sites: Luria's game, DCCS, Forward span, Backward span, Verbal fluency. These tasks were preceded by a check to make sure children recognized the pictures on the DCCS stimuli (all tested participants did so) and pilot theory of mind tasks, data from which we do not report here.

Performance was recorded on an Android tablet through the Kobo platform. In the UK sample the experimenter both conducted the study and recorded the results. In all other samples a bilingual RA conducted the study in the local language (Tsiman in the Bolivian group, Herero in the Kunene groups) while the experimenter recorded children's responses on the tablet. Below we will refer to the "researcher" as whichever person was directly administering the study. All tasks were translated and back-translated at least twice in both non-UK contexts to ensure accuracy and comprehension.

We provide a full PDF of the battery as it appeared on the tablet screen, including verbatim scripts for the researcher for every task in the OSF repository. Here we provide stimuli and additional details for each task which are not included in that document.

Luria's game

This task involved 15 imitation trials (e.g. if the researcher showed a fist the participant did the same) followed by 15 counter-imitation trials (e.g. if the researcher showed a palm the participant showed a fist). For both imitation and counter-imitation trials there were three randomized orders in which the researcher displayed fist and palm. Which of the three orders a given participant saw was decided randomly by the Kobo program.

Dimensional change card sort (DCCS)

We used the following images in the DCCS task for the target cards and cards the child sorted into boxes:

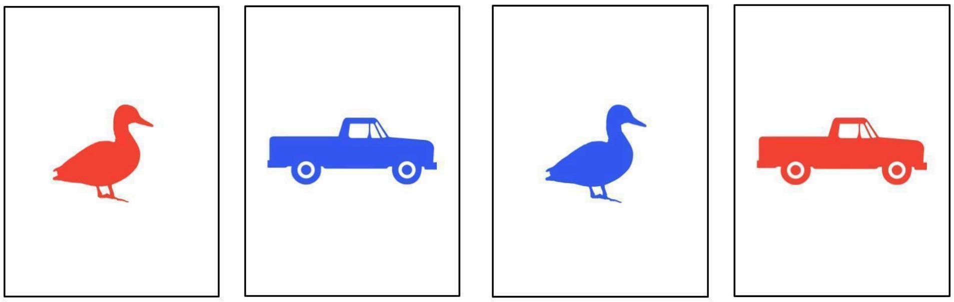

The order of pre-switch and post-switch trials was semi-randomized in the same way as Luria's task orders: In each case there were three pre-set randomized orders for pre-switch and three for post-switch and which of these were seen by any participant was randomized.

Forward and backward span

While forward and backward span tasks typically use numbers as stimuli, we judged this inappropriate for our sample since 1) familiarity with number words was liable to differ between schooled and unschooled groups and 2) the syllable length of number words differs dramatically across languages (e.g. in English the list of words for numbers 1-10 contains eleven syllables, in Herero, the language of our Kunene sample, the same list contains twenty-seven syllables). Instead, we used common, two- or three-syllable nouns in all groups (e.g. 'chicken', 'table',

‘apple’) for both span tasks. Participants were given lists of words to repeat (forwards or backwards for the respective tasks) until they made an error on two consecutive lists. The longest list they completed prior to these errors was marked as their forward/backward span score.

Verbal fluency

The typical categories used for verbal fluency tasks are animals and foods, assumed to be topics on which children have particularly extensive knowledge of nouns. Given that cultures differ significantly in the volume of known words for different categories, however, we identified categories at each site for which children would be likely to know most items. In the UK we followed established practice and used animals and foods. In the Kunene groups, preliminary ethnographic work suggested that these same categories would be at least as well-known by children as any other. Similar preliminary work in the Bolivian context revealed that types of fish was a particularly salient and well-known category, which we used instead of foods.

Estimating ages in non-UK samples

In some cases in the Bolivian and Kunene samples, children’s ages were known by the child, their parents, teachers or peers. In those cases when they were not ages were estimated by at least two researchers with local experience, comparing children to peers whose ages were known.

Details of rates at which comprehension checks were passed

In DCCS, all children in the UK sample successfully matched cards on the first dimension (pre-switch trials) and were thus judged to understand the task. In the Bolivian, schooled and unschooled Kunene samples 7, 3 and 6 children failed our checks, respectively (9%, 4% and 10%). No participants failed to understand Luria’s game or forward span - in all cases all children performed the correct behaviors (i.e. counter-imitating gestures, reciting words from the given list). All children in the UK and schooled Kunene groups understood verbal fluency, and only two children in each of the unschooled Kunene and Bolivian samples (3% in each case) failed to produce any words from the relevant category. In sum, comprehension of EF tasks was 90% or above in all cases and generally at or near ceiling. All participants who did not pass our ‘task demand checks’ were excluded from the data (again with the exception of Backwards Span, discussed below).

Details of schooling situation in Tsimaneland

The quality of the Bolivian schools in the regions we sampled was limited by a number of structural factors (which were absent in Kunene). Most notably, there was typically absence of a common language between students and teachers: Students typically speak only Tsiman, but teachers speak only Spanish as a result of recent government reforms requiring formal qualifications typically inaccessible for local (Tsiman-speaking) educators. The Tsimane are swidden horticulturalists residing in densely-wooded areas of lowland Bolivia, often far from urban areas and teacher-training centers. As a result, while students attended regularly in order to receive government subsidies, the effect of actual instruction appeared limited and teachers often resorted to having students repeat rhythms they banged out on desks, apparently for lack of a better form of communication. To reflect this schooling experience we will refer to the Bolivian group as having ‘limited schooling’ and the Kunene group (where schooling had no equivalent issues) as having ‘high-quality schooling.’

Study populations: sample characteristics and demographics

Bolivia

Data were collected in 2021 in the Amazonian River Basin of Bolivia among the Tsimane people. The sample included 73 children (43 boys, 30 girls) from age 5 to 17 in three communities. One of the authors (HD) has worked in these communities included in this study for over 10 years, and she has done extensive ethnographic work on social learning and schooling (59, 40). Children's age was confirmed by cross-validating interview responses with an extensive population register collected and continuously updated since 2002 by the Tsimane Health and Life History Project, one of the longest health and anthropology projects within a subsistence-based society to date (39). Age estimations rely on in-depth demographic interviews, relative age lists determined from reproductive histories, hospital records, and baptism records (see 39 for more details)

The Tsimane are from the same ethnolinguistic group, making them culturally and linguistically homogeneous relative to industrialized populations. Though Tsimane children begin attending school sometime between 5-7 years old, Tsimane children who live in the villages do not grow up in a schooled world. For example, attending school is generally children's first exposure to the written alphabet and counting beyond small numbers that describe aspects of their immediate surroundings (e.g. one capuchin monkey, two stars in the sky), and by 5th grade children have attended 50% fewer hours of school compared to children in the US and Europe (40). Because of limited material wealth inequality, Tsimane children's schooling exposure or the quality of school they attend is not confounded by family wealth.

Our work was supported by extensively trained bilingual Tsimane research assistants, in particular one of the authors on this paper (ABC) who went to great lengths to ensure the comprehension and proper translation of all of the tasks. (Through extensive translation, back-translation, piloting etc.) Both HD and ABC have close and longstanding connections across the Tsimane communities where this work was conducted.

Kunene region (Namibia/Angola) - schooled and unschooled samples

The present work was conducted across both sides of the Namibia/Angola border in 2022 as part of the Ecology of Mind Research Project, developed and maintained by two of the authors (IK,HD). Across the entire sample there were 134 children who participated in the study between the ages of 5 and 18. Communities in Namibia were selected because those communities had already participated in ongoing demographic and ethnographic data collection for over a decade (60, 61). In Angola, HD began collecting comparable demographic and ethnographic data in 2019. Ages of participants were collected before testing and cross-validated with parents or other family members and caregivers. If children were attending schools, ages could be cross-validated with school records or with teachers.

Participants in the Kunene sample came from three different groups (Himba, Twa, Zemba). These groups, and several others in the area, are closely-related Bantu tribes who share mutually intelligible dialects, cultural practices and even matriclans (62, 61). Moreover, the groups share a common lingua franca, Herero, in which all individuals are fluent and in which all testing was conducted.

Some children of all three groups attend school in Namibia. Our schooled Kunene sample was drawn from this group. Specifically, from rural schools - such that, outside of the schoolroom children's experience was not shaped by a broader schooled world (e.g. regularly using money, interfacing with government institutions, having access to market goods outside of school materials etc.) In the case of this study, children from all three groups in Namibia live in either the same or nearby villages, attend the same schools, and today have similar economic and marriage practices.

Though we are aware of no published ethnographies which concern all three of the tested populations together, details regarding relevant similarities can be found in ethnographies focused on a single group or in previous studies conducted in the same communities (e.g. 59, 61, 62).

Schools teach in Herero - the common language of the region in which schooling is also conducted for the first several grades and that all children attend these classes and understand because Herero is also a Bantu dialect. The composition of the schooled and non-schooled sample was similar in terms of the three tribal groups, with a majority of children being Himba and minorities Twa and Zemba. As such, there is no reason to suspect that differences in tribal affiliation contribute to group differences reported here.

Data collection was supported by expert bilingual research assistants, in particular, Vehamburuka Hartley, an author on this paper, who significantly contributed to her contributions in translations and back-translations, piloting, and running the tasks. In all cases, utmost care was taken to abide by and respect local customs. Prior to testing in any location, community leaders (headmen, school principals) were consulted and their permission was acquired. In most cases the experimenters, or their close colleagues who were also present at the time, had longstanding relationships with the communities/schools in which the studies were conducted.

UK

Testing in the UK occurred as part of Nottingham University's "Summer Scientist Week" in which children of all ages are invited to participate in a range of studies at the university. 76 children, ages 3 to 15 (42 boys, 34 girls) participated in the study. Ages of all participants were verified with parents prior to attending the event. All children in accordance with UK law, children attend school from five years of age. This means all children in our sample, with the exception of one three-year-old, were already in or about to enter school. Moreover, since the UK is certainly a schooled world, it is almost certain that all children in this sample were exposed to school-like cognition and materials (e.g. reading, math) from infancy onwards via parents and/or pre-school.

As we discuss in the main text, this reflects a typical approach to sampling in developmental cognitive science, which is specifically what we require for the purposes of this study (i.e. comparing a typical cognitive science sample to groups not usually studied in mainstream EF research).

Developing and interpreting measures

In the UK sample, EF measures were deployed in English following standard protocols for each task.

In the Bolivian and Kunene samples, EF tasks went through half a dozen or more rounds of translation and back-translation in order to ensure the exact wording was correct and comprehensible to local participants. Pilot studies were conducted at both sites to ensure children in each group could understand the tasks and pass comprehension checks. Throughout, task development was informed by local experts (ABC and VH, as well as other researchers and assistants at the fieldsites).

Testing environment and participant reactions

At all sites children were tested one at a time with no peers or adults present in the immediate vicinity (approx three feet) of the testing table (though in all cases parents and peers were typically present immediately beyond this boundary). All individuals present were instructed not to help or comment on the participants' performance during the tasks.

In the Bolivian and Kunene samples, the tasks were administered by local research assistants, while the experimenter (IK) recorded children's responses on a tablet. In the Bolivian and schooled Kunene sample testing was conducted on school grounds, either in an empty classroom or building, or outside, adjacent to classroom walls. In the non-schooled samples, a research area was created in or near a community, allowing children to participate in familiar and comfortable surroundings while still being private. This either took the form of a large tent with a

table with three chairs (one for the participant and one each for experimenter and research assistant), or the same table and three chairs placed outside in the shade.

In the UK sample the experimenter (IK) both administered the tests and recorded performance. Testing occurred on University of Nottingham premises in a large room where other, unrelated, studies were occurring in parallel some 20-30 feet away. In sum, in all cases children were tested in quiet but not silent environments, typically with some degree of noise from their peers audible in the near distance. This was never a distraction to the children (indeed in our experience children are remarkably tolerant to social noise, e.g. readily focusing on developmental tasks when being tested in noisy children's museum settings).

In all cases, children were first familiarized with the individual(s) running the study — in those cases where they did not already know them (as often happened in Bolivia and Kunene). The research assistant and experimenter had a brief friendly conversation with each child and explained that they would be playing some games with them prior to testing. As we discuss in detail in the main text, each task contained some control for whether participants understood the basic instructions of the task. Specifically, controls for each task were: DCCS and Luria's game - passing pre-switch trials, forward and backward span - recalling at least two words forward from the list, verbal fluency - naming at least two things from the correct category. Data from children who did not pass these controls was not included in the reported results.

Reactions to particular tasks (qualitative reports)

Luria's hand game, VF and Forward span: Participants across groups were engaged by the task and often visibly amused and frustrated (often laughing) by their inability to recall more words or making errors in the hand game.

Backward span: Participants who recalled some items in reverse order reacted to the task similarly as forward span (i.e. often frustrated they were not able to recall more words). Those who did not recall even two words in reverse order, unsurprisingly, expressed confusion and typically continued to repeat the words in the same order.

DCCS: Participants in all groups patiently sorted cards and listened to instructions. In neither the UK sample nor those from outside of schooled worlds did children express confusion regarding the switch in dimensions. In this context it is important to note, again, that the instructions for switching were repeatedly slowly and twice in full detail, leaving the participant ample time to 'process' what was being asked.

In sum, while the context of such tasks was certainly less familiar to Tsimane and Kunene-region participants than to UK schoolchildren, we observed no differences in response to the tasks which would suggest a qualitative difference in how children approached them. This further supports the quantitative result that children in all groups performed as expected in the comprehension checks for every task. Again the key claim here is that performance across groups reflects the tasks *as they are designed and routinely administered*. It is only in this way that we can achieve our set goal of obtaining data on task performance (*not* underlying constructs) which is comparable across population.

Additional regression tables

Table S1. Main model (Tables 1 and 2, main text) with Bolivia as reference group.

	Dependent variable:			
	DCCS	Luria's test	Verbal Fluency	Forward span
	logistic	*normal*	*normal*	*normal*
	(1)	(2)	(3)	(4)
age	−0.344***	0.352***	1.106***	0.080***
	(0.068)	(0.071)	(0.102)	(0.015)
locationBritish	−2.567***	1.742**	15.032***	0.904***
	(0.502)	(0.614)	(0.875)	(0.126)
locationSchooled Kunene	−1.715**	0.179	1.831*	−0.345**
	(0.529)	(0.598)	(0.845)	(0.123)
locationUnschooled Kunene	0.381	−1.242*	0.196	−0.469***
	(0.402)	(0.610)	(0.870)	(0.125)
Constant	2.992***	7.396***	−0.900	2.628***
	(0.712)	(0.860)	(1.245)	(0.177)
Observations	268	284	279	283
Log Likelihood	−115.850	−760.694	−842.064	−309.429
Akaike Inf. Crit.	241.700	1,531.389	1,694.128	628.857

*p<0.05; **p<0.01; ***p<0.001

Table S2. Main model (Tables 1 and 2, main text) with Unschooled Kunene as reference group.

	Dependent variable:			
	DCCS	Luria's test	Verbal Fluency	Forward span
	logistic	*normal*	*normal*	*normal*
	(1)	(2)	(3)	(4)
age	−0.344***	0.352***	1.106***	0.080***
	(0.068)	(0.071)	(0.102)	(0.015)
locationSchooled Kunene	−2.097***	1.422*	1.635	0.124
	(0.533)	(0.630)	(0.891)	(0.129)
locationBolivia	−0.381	1.242*	−0.196	0.469***
	(0.402)	(0.610)	(0.870)	(0.125)
locationBritish	−2.948***	2.985***	14.836***	1.373***
	(0.508)	(0.615)	(0.876)	(0.126)
Constant	3.373***	6.154***	−0.703	2.159***
	(0.719)	(0.819)	(1.184)	(0.168)
Observations	268	284	279	283
Log Likelihood	−115.850	−760.694	−842.064	−309.429
Akaike Inf. Crit.	241.700	1,531.389	1,694.128	628.857

*p<0.05; **p<0.01; ***p<0.001

Table S3. Models for DCCS pass/fail status regressed on age used to generate the curves in Figure 2.

	Dependent variable:			
	DCCS pass/fail			
	UK	Schooled Kunene	Unschooled Kunene	Bolivia
age	0.100	0.900^{**}	0.258^{**}	0.334^{***}
	(0.288)	(0.393)	(0.109)	(0.104)
Constant	2.436	-5.894^{*}	-2.533^{**}	-2.898^{***}
	(2.200)	(3.089)	(1.099)	(1.038)
Observations	76	67	58	66
Log Likelihood	−12.572	−12.572	−36.921	−37.608
Akaike Inf. Crit.	29.144	29.143	77.842	79.215
Note:				*p<0.1; **p<0.05; ***p<0.01

Table S4. Models for recalling greater than four/less than or equal to four items in forward span on age used, to generate the curves in Figure 3, left panel.

	Dependent variable:			
	Forward span greater than four			
	UK	Schooled Kunene	Unschooled Kunene	Bolivia
age	0.229**	0.025	0.149	0.135
	(0.111)	(0.156)	(0.322)	(0.220)
Constant	−2.405***	−3.099	−5.679	−5.096*
	(0.923)	(1.938)	(3.678)	(2.728)
Observations	76	70	64	73
Log Likelihood	−47.189	−15.319	−5.049	−8.974
Akaike Inf. Crit.	98.378	34.639	14.098	21.948
Note:				*p<0.1; **p<0.05; ***p<0.01

Table S5. Models for backwards span pass/fail (i.e. able to recall at least two items in reverse order) regressed on age, used to generate the curves in Figure 3, right panel. UK model not included since all participants succeeded.

	Dependent variable:		
	Backward span pass/fail		
	Schooled Kunene	Unschooled Kunene	Bolivia
age	0.789**	0.498***	0.425***
	(0.333)	(0.146)	(0.105)
Constant	−4.878*	−6.523***	−4.504***
	(2.629)	(1.653)	(1.131)
Observations	70	64	73
Log Likelihood	−13.498	−25.036	−38.640
Akaike Inf. Crit.	30.997	54.073	81.280
Note:			*p<0.1; **p<0.05; ***p<0.01

Table S6. Regressions for DCCS, Forward span and Backwards span binomial measures (pass/fail, more/fewer than four items, pass/fail, respectively) with location and location-by-age interaction terms. UK used as a reference group.

	Dependent variable:		
	DCCS, pass/fail	Forward span, more than 4 items	Backward span, pass/fail
	(1)	(2)	(3)
Age	0.100	0.229**	−0.00000
	(0.288)	(0.111)	(547.935)
Schooled Kunene	−5.335**	−2.690	−24.070
	(2.432)	(2.880)	(4,435.886)
Unschooled Kunene	−4.970**	−3.274	−26.089
	(2.459)	(3.792)	(4,435.886)
Bolivia	−8.330**	−0.694	−24.444
	(3.793)	(2.146)	(4,435.887)
Age*Schooled Kunene	0.234	−0.093	0.425
	(0.307)	(0.247)	(547.935)
Age*Unschooled Kunene	0.158	−0.080	0.498
	(0.308)	(0.341)	(547.935)
Age*Bolivia	0.800	−0.204	0.789
	(0.488)	(0.191)	(547.935)
Constant	2.436	−2.405***	19.566
	(2.200)	(0.923)	(4,435.886)
Observations	267	283	283
Log Likelihood	−99.672	−76.532	−77.175
Akaike Inf. Crit.	215.344	169.064	170.350
Note:			*p<0.1; **p<0.05; ***p<0.01

Table S7. Regressions for DCCS, Forward span and Backwards span binomial measures (pass/fail, more/fewer than four items, pass/fail, respectively) with location and location-by-age interaction terms. Schooled Kunene used as a reference group.

	Dependent variable:		
	DCCS, pass/fail	Forward span, more than 4 items	Backward span, pass/fail
	(1)	(2)	(3)
Age	0.900**	0.025	0.789**
	(0.393)	(0.156)	(0.333)
British	8.330**	0.694	24.444
	(3.793)	(2.146)	(4,435.887)
Unschooled Kunene	2.995	−1.997	0.374
	(3.259)	(3.347)	(2.863)
Bolivia	3.360	−2.580	−1.645
	(3.279)	(4.157)	(3.106)
Age*British	−0.800	0.204	−0.789
	(0.488)	(0.191)	(547.935)
Age*Unschooled Kunene	−0.566	0.111	−0.364
	(0.407)	(0.270)	(0.349)
Age*Bolivia	−0.642	0.124	−0.291
	(0.408)	(0.358)	(0.363)
Constant	−5.894*	−3.099	−4.878*
	(3.089)	(1.938)	(2.630)
Observations	267	283	283
Log Likelihood	−99.672	−76.532	−77.175
Akaike Inf. Crit.	215.344	169.064	170.350
Note:			*p<0.1; **p<0.05; ***p<0.01

Table S8. Regressions for DCCS, Forward span and Backwards span binomial measures (pass/fail, more/fewer than four items, pass/fail, respectively) with location and location-by-age interaction terms. Unschooled Kunene used as reference group.

	Dependent variable:		
	DCCS, pass/fail	Forward span, more than 4 items	Backward span, pass/fail
	(1)	(2)	(3)
Age	0.258**	0.149	0.498***
	(0.109)	(0.322)	(0.146)
Schooled Kunene	−3.360	2.580	1.645
	(3.279)	(4.157)	(3.106)
British	4.970**	3.274	26.089
	(2.459)	(3.792)	(4,435.886)
Bolivia	−0.365	0.583	2.019
	(1.512)	(4.580)	(2.003)
Age*Schooled Kunene	0.642	−0.124	0.291
	(0.408)	(0.358)	(0.363)
Age*British	−0.158	0.080	−0.498
	(0.308)	(0.341)	(547.935)
Age*Bolivia	0.077	−0.014	−0.073
	(0.151)	(0.390)	(0.180)
Constant	−2.533**	−5.679	−6.523***
	(1.099)	(3.678)	(1.653)
Observations	267	283	283
Log Likelihood	−99.672	−76.532	−77.175
Akaike Inf. Crit.	215.344	169.064	170.350
Note:			*p<0.1; **p<0.05; ***p<0.01

Table S9. Regressions for DCCS, Forward span and Backwards span binomial measures (pass/fail, more/fewer than four items, pass/fail, respectively) with location and location-by-age interaction terms. Bolivia used as a reference group.

	Dependent variable:		
	DCCS, pass/fail	Forward span, more than 4 items	Backward span, pass/fail
	(1)	(2)	(3)
Age	0.334***	0.135	0.425***
	(0.104)	(0.220)	(0.105)
Unschooled Kunene	0.365	−0.583	−2.019
	(1.512)	(4.580)	(2.003)
Schooled Kunene	−2.995	1.997	−0.374
	(3.259)	(3.347)	(2.863)
British	5.335**	2.690	24.070
	(2.432)	(2.880)	(4,435.886)
Age*Unschooled Kunene	−0.077	0.014	0.073
	(0.151)	(0.390)	(0.180)
Age*Schooled Kunene	0.566	−0.111	0.364
	(0.407)	(0.270)	(0.349)
Age*British	−0.234	0.093	−0.425
	(0.307)	(0.247)	(547.935)
Constant	−2.898***	−5.096*	−4.504***
	(1.038)	(2.729)	(1.131)
Observations	267	283	283
Log Likelihood	−99.672	−76.532	−77.175
Akaike Inf. Crit.	215.344	169.064	170.350
Note:			*p<0.1; **p<0.05; ***p<0.01

Scatterplots

Figure S1. Scatterplot showing children's performance on the DCCS task across the four study populations. Data points are jittered to improve visibility.

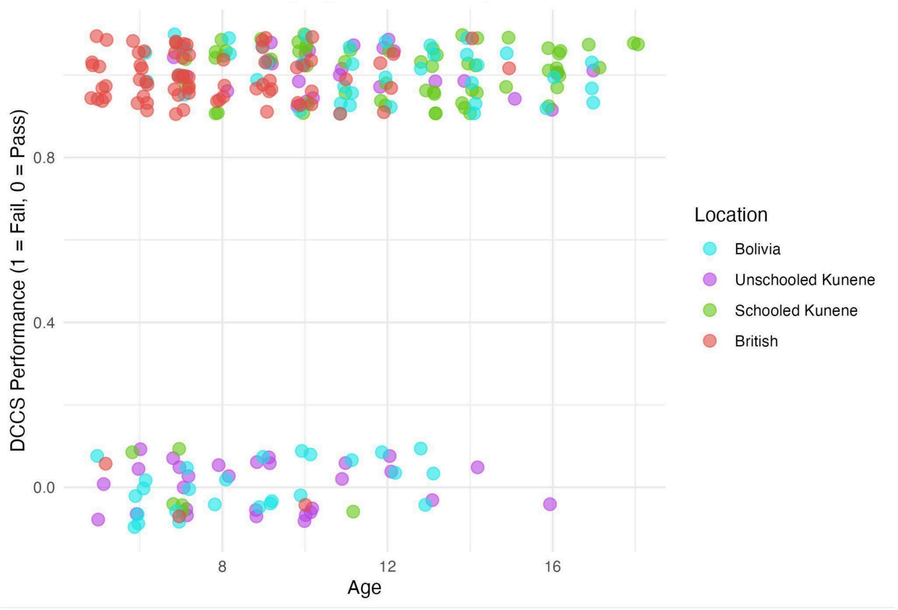

Figure S2. Scatterplot showing children's performance on the Luria's test across the four study populations. Data points are jittered to reduce overlap.

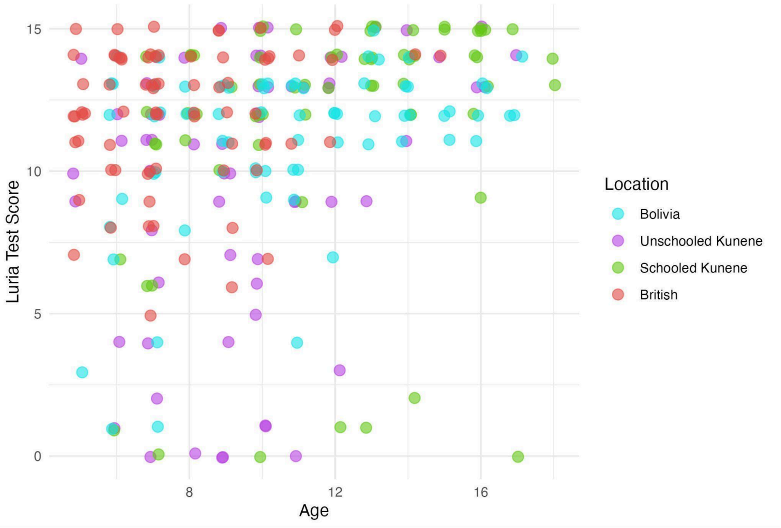

Figure S3. Scatterplot showing children's performance on the Verbal fluency task across the four study populations. Data points are jittered to reduce overlap.

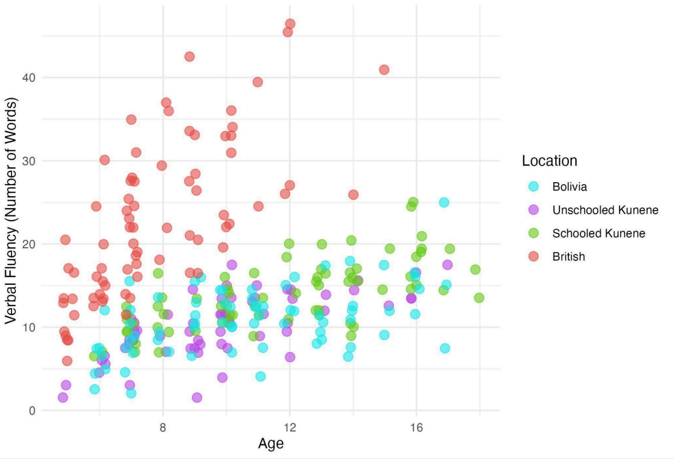

Figure S4. Scatterplot showing children's performance on forward span across the four study populations. Data points are jittered to reduce overlap.

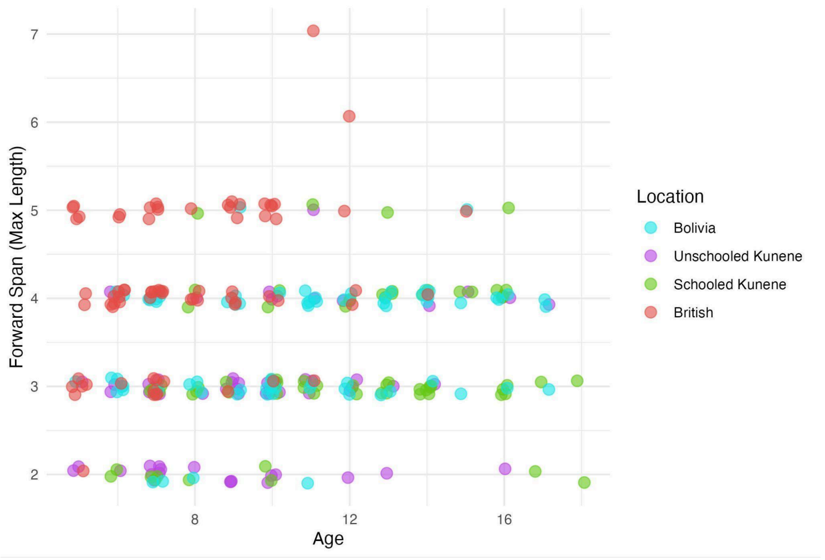

Figure S5. Scatterplot showing children's performance on backward span across the four study populations. Data points are jittered to reduce overlap.

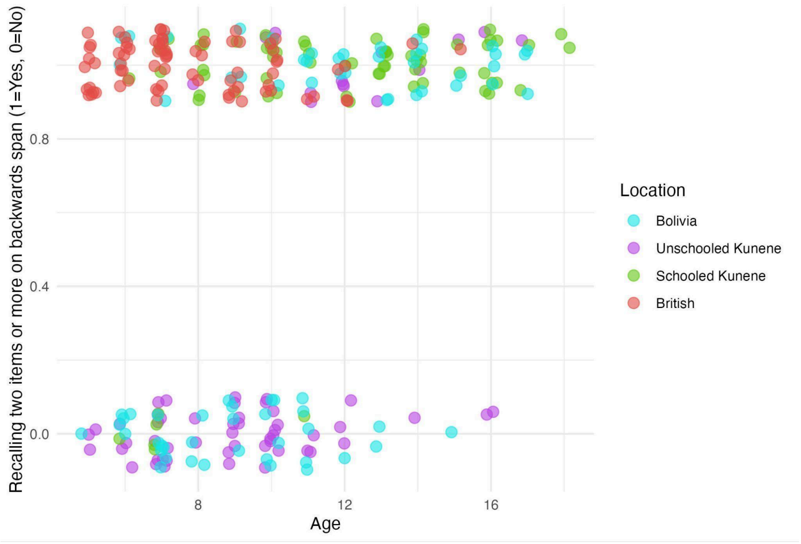

Dataset S1 (separate file). Available at https://osf.io/8uz9s/

R code S1 (separate file). Available at https://osf.io/8uz9s/
